# Modelling the relative precision of whole-kidney dosimetry in molecular radiotherapy using a power law approach

**DOI:** 10.1093/rpd/ncaf123

**Published:** 2026-03-13

**Authors:** Jehangir Khan, Tobias Rydén, Martijn van Essen, Johanna Svensson, Esmaeil Mehrara, Magnus Båth, Peter Bernhardt

**Affiliations:** Department of Medical Physics, Faculty of Medicine and Health, Örebro University Hospital, 70185 Örebro, Sweden; Department of Medical radiation Sciences, Institute of Clinical Sciences, Sahlgrenska Academy at University Gothenburg, SE-41345 Gothenburg, Sweden; Department of Biomedical Engineering and Medical Physics (MFT), Sahlgrenska University Hospital, Gothenburg, SE-41345 Gothenburg, Sweden; Department of Clinical Physiology, Sahlgrenska University Hospital, SE-41345 Gothenburg, Sweden; Department of Molecular and Clinical Medicine, Institute of Medicine, Sahlgrenska Academy at University of Gothenburg, SE-41345 Gothenburg, Sweden; Department of Oncology, Institution of Clinical Sciences, Sahlgresnka Academy at Universiy of Gothenburg, SE-41345 Gothenburg, Sweden; Department of Medical radiation Sciences, Institute of Clinical Sciences, Sahlgrenska Academy at University Gothenburg, SE-41345 Gothenburg, Sweden; Department of Biomedical Engineering and Medical Physics (MFT), Sahlgrenska University Hospital, Gothenburg, SE-41345 Gothenburg, Sweden; Department of Medical radiation Sciences, Institute of Clinical Sciences, Sahlgrenska Academy at University Gothenburg, SE-41345 Gothenburg, Sweden; Department of Biomedical Engineering and Medical Physics (MFT), Sahlgrenska University Hospital, Gothenburg, SE-41345 Gothenburg, Sweden; Department of Medical radiation Sciences, Institute of Clinical Sciences, Sahlgrenska Academy at University Gothenburg, SE-41345 Gothenburg, Sweden; Department of Biomedical Engineering and Medical Physics (MFT), Sahlgrenska University Hospital, Gothenburg, SE-41345 Gothenburg, Sweden

## Abstract

A power-law model was introduced to characterize the relationship between spherical volumes-of-interest (SVs) and whole kidney parenchyma (WKP)-derived absorbed dose estimates, enabling quantitative precision for image-based dosimetry. Single photon emission computed tomography/computed tomography (SPECT/CT) images were acquired at 24, 48, and 168 h after [^177^Lu]Lu-DOTATATE treatment in 18 patients. Kidney activity was quantified using WKP and SV-based methods (2 and 0.6 ml) on SPECT-images. WKP and both SV approaches showed good agreement in kidney dosimetry, with normalization factors of 1.12 and 1.23 (standard error mean ≤1.2%), and improved precision when multiple SVs were used. The power-law model demonstrated excellent fit (*R*^2^ > 0.97) and high precision (~7%), with no significant difference between SV sizes (*P* = .15) and minimal bias (<0.003%). This power law model presents a novel method for quantifying the relative precision of SPECT-derived kidney dosimetry. Further validation is warranted to address residual uncertainty and complex noise correlation in the SV-based dose estimates.

## Introduction

Radiolabeled somatostatin analogues, such as [^177^Lu]Lu-DOTATATE, have shown promising results in the treatment of metastasized or inoperable neuroendocrine tumours (NETs) [1–3]. The NETTER-1 trial demonstrated improved progression-free survival in NET patients treated with [^177^Lu]Lu-DOTATATE [4]. However, inter-patient variability in the estimated absorbed doses to both healthy organs and tumours has been reported [5–8], highlighting the importance of personalized dosimetry to optimize therapeutic outcomes. Consequently, several hospitals and research institutes across Europe recommended the integration of dosimetry into routine molecular radionuclide therapy (MRT) to optimize personalized treatment [9].

Accuracy in quantitative SPECT (single photon emission computed tomography) imaging and estimation of the mean absorbed dose in a volume of interest (VOI), such as the whole kidney parenchyma (WKP), remains a significant challenge [10–13]. This difficulty is further amplified when relying on measurements from only a subset of voxels, as is performed with a small volume of interest (SVs). These voxel-level measurements are inherently noisy, and the precision of dose estimate SV$(k)$ depends on the number of small volumes *k*, their spatial distribution, and the quality of the underlying SPECT image.

It is important to distinguish between precision and bias when describing the uncertainties inherent to SPECT-based dosimetry. Precision refers to the reproducibility of the measurements under repeated sampling, primarily reflecting random noise. In contrast, bias represents systematic deviations, such as consistent over- or underestimation. In this study, we focused exclusively on the quantification of measurement precision.

While previous studies have assessed the precision of the WKP method using phantom data and simulations [14], we propose a generalizable power-law model [15, 16] that characterizes the relationship between the SV and WKP estimates. This model enables the estimation of WKP precision based solely on SV data alone. Though empirical, the approach is grounded in statistical theory and provides a practical, robust framework for evaluating precision in imaging-derived dosimetry.

## Materials and methods

### Patients

This study was carried out on a sample of 18 (male = 8, female = 10) NETs patients who received [^177^Lu]Lu-DOTATATE (Lutathera®, AAA) treatment at Sahlgrenska University Hospital between 2019 and 2021. The average age of the patients at the time of treatment was 71 y (range: 56–86 y). The Swedish Ethical Review Authority approved the study, waiving the requirement of consent to participate (2020-05092).

To reduce radiation exposure to the kidneys, patients were administered an amino acid solution (Vamin® 14 gN/l. 2000 ml Fresenius Kabi AG, Bad Homburg, Germany) at an infusion rate of 400 ml/h, starting 30 min prior to [^177^Lu]Lu-DOTATATE administration during each treatment cycle. The average administered activity of [^177^Lu]Lu-DOTATATE was 7.5 GBq (range: 7.4–7.6 GBq).

### Data acquisition and image reconstruction

SPECT and CT (computed tomography) imaging data were acquired using a hybrid SPECT/CT system (Discovery NM/CT 670, GE Healthcare, Waukesha, WI, USA). Imaging of the upper abdomen was performed ~24, 48, and 168 h post injection (h. p. i). The SPECT system used an NaI (Tl) crystal (5/8″) and a medium-energy parallel-hole collimator. The acquisition parameters included a 208 keV ±10% energy window, 60 projections (30 s per projection), and 128 × 128 matrix with 4.42 mm slice thickness and pixel size. CT scans were acquired with a 120 kV tube voltage, a noise index of 27.5, and activated dose modulation. CT images were reconstructed using a 512 × 512 matrix with a slice thickness of 5 mm.

SPECT projections were reconstructed using in-house image analysis and reconstruction platform PhONSAi 4.0 [17]. All SPECT images were reconstructed with attenuation-, scatter-, and collimator detector response correction using ordered subset expectation maximization reconstructions (ASCC-OSEMs) based on the Monte-Carlo code SARec [18]. SPECT data reconstruction was performed with 10 subsets and 6 iterations, followed by post-filtering with a Gaussian filter (GF), applying sigma values ranging from 0 to 6 mm.

### Dosimetry workflow

The absorbed dose to the kidneys was evaluated based on the acquired SPECT/CT images. Activity concentrations within volumes of interest were determined from the SPECT images using two methods: WKP and SVs with volumes of 2 ml (SV_2_), and 0.6 ml (SV_0.6_), placed within representative regions of the WKP. WKP segmentation was performed on CT images acquired at 24, 48, and 168 h. p. i. of [^177^Lu]Lu-DOTATATE, and then propagated to the corresponding SPECT images, as previously described [19]. To correct for misregistration caused by breathing-induced motion between CT and SPECT acquisitions, CT-based WKP segmentations were adjusted on SPECT images [20]. This adjustment ensured that the kidney parenchyma activity on SPECT fell within the boundaries of the segmented WKP drawn on CT. This process accounts for the possible translational and rotational motion of both kidneys during the scan.

Activity concentration (MBq/g) for each VOI and time point was determined using counts per second (cps). The SPECT calibration factor was determined using a Jaszczak phantom filled with known activity, following a previously described protocol [19].

To evaluate the small VOI method, up to 5 SVs of volumes 2 ml, or 0.6 ml were placed within each kidney parenchyma. These SVs were positioned to reflect the overall distribution of radioactivity in the WKP, typically near the kidney pelvis and slightly away from the outer cortex. Activity concentrations in each SV were calculated as previously described [19]. The measured activity concentrations were corrected for partial volume effects (PVEs) using patient-specific kidney geometries. The recovery coefficients (RCs) were determined for each kidney (left and right) using segmented WKPs from CT images at 24, 48, and 168 h. p. i. The SARec algorithm was used to simulate SPECT data based on CT-based segmentation filled with known activity, using the same acquisition parameters as in clinical imaging. The normalization factor for the SV method (NF_SV_) was then calculated relative to that of the WKP method, as previously outlined [19].


1 \begin{eqnarray*} {\mathrm{NF}}_{\mathrm{SV}}=\frac{\sum_{k=1}^n\frac{{\mathrm{D}}_{\mathrm{SV}}}{{\mathrm{D}}_{\mathrm{WKP}}}}{n} \end{eqnarray*}

where *n* is the total number of individual SVs evaluated across all kidneys. In this study, *n* = 36 kidneys × 5 SVs, with a total of 180 data points were used to calculate the mean NF_SV_. The kidney absorbed doses, ${\mathrm{D}}_{\mathrm{SV}}$ and ${\mathrm{D}}_{\mathrm{WKP}}$, were estimated using the SV and WKP methods, respectively.

### Application of precision modelling

The precision of WKP-derived kidney absorbed dose estimates was evaluated by developing a statistical noise modelling approach-based on SV measurements. This section focuses on understanding how statistical fluctuations and image-based noise influence absorbed dose estimates. Specifically, we use SV-based measurements to model the variance and correlation structure relative to the WKP reference. This modelling enables a quantitative evaluation of the expected precision gains from averaging multiple SVs and provides a theoretical basis for the power-law behaviour observed in the data.

### Precision modelling based on SV and WKP comparison

The underlying, noise-free absorbed dose in the kidney is denoted as D_true_. Each single SV measurement of the kidney absorbed dose is assumed to be a noisy observation of D_true_ that is,


2 \begin{eqnarray*} {\mathrm{D}}_{\mathrm{S}{\mathrm{V}}_i}={\mathrm{D}}_{\mathrm{true}}+{\varepsilon}_i \end{eqnarray*}

where ${\varepsilon}_i$ is a random noise term with a zero mean and variance ${\sigma^2}_{\mathrm{SV}}$. In practice, each SV is also subjected to normalization against the WKP. This normalization minimizes the bias between the methods but introduces population-level variance into the SV measurements, contributing to ${\sigma^2}_{\mathrm{SV}}$.

The mean of *k* such SVs is:


3 \begin{eqnarray*} {{\mathrm{D}}_{\mathrm{SV}}}_{\mathrm{mean}}(k)=\frac{1}{k}\times{\sum}_{n=1}^k\left({{\mathrm{D}}_{\mathrm{SV}}}_i\right)={\mathrm{D}}_{\mathrm{true}}+\overline{\varepsilon_k} \end{eqnarray*}

where $\overline{\varepsilon_k}$ has a variance *σ*^2^_SV_/*k*. Thus, the variance of the estimate is proportional to 1/*k*, which is a classical result of the central limit theorem.

The WKP estimate of the absorbed dose in the kidney was computed as the mean of a larger number of voxels, or SVs, within the kidney. This can be represented as


4 \begin{eqnarray*} {\mathrm{D}}_{\mathrm{WKP}}={\mathrm{D}}_{\mathrm{true}}+{\varepsilon}_{\mathrm{WKP}} \end{eqnarray*}

where ${\varepsilon}_{\mathrm{WKP}}$ is a noise term with variance ${\sigma^2}_{\mathrm{WKP}}$, arising from image noise, segmentation errors, patient and kidney movement during image acquisition, and preprocessing corrections, such as partial volume correction (PVC).

### Correlation between SV_mean_(*k*) and WKP

${\mathrm{SV}}_{\mathrm{mean}}(k)$ and WKP are not independent estimates, as both are derived from the same tissue and incorporate the identical noise-free absorbed dose signal D_true_ but have distinct noise components. The correlation *ρ* between them reflects this shared origin.

▪ If ${\mathrm{SV}}_{\mathrm{mean}}(k)$and WKP are based on disjointed voxel sets, their noise terms are uncorrelated.
▪ If they overlap or sample a spatially autocorrelated field, the noise terms are partially correlated.
▪ If the WKP is noise-free or perfectly aligned with ${\mathrm{SV}}_{\mathrm{mean}}(k),$the noise is fully correlated, and the imprecision vanishes.

In practice, SVs are randomly sampled within the WKP but without exact overlap. Due to the spatial autocorrelation in the imaging data, the noise components of the SV and WKP estimates remain partially correlated.

To isolate the measurement precision from the biological variability of the absorbed dose, we considered the relative difference (RD) between the SV and WKP methods. If both are proportional to D_true_ with some multiplicative noise, the RD becomes:


5 \begin{eqnarray*} \mathrm{RD}\ \left(\%\right)=\left(\frac{{\mathrm{SV}}_{\mathrm{mean}}-\mathrm{WKP}}{\mathrm{WKP}}\right)\times 100 \end{eqnarray*}

In this formulation, D_true_ cancels out and RD depends only on the measurement noise. This enables the assessment of precision independent of absolute dose values.

To further justify the modelling approach, consider the generative model:


6 \begin{eqnarray*} {\mathrm{X}}_{\mathrm{SV}}={\mathrm{D}}_{\mathrm{true}}\times \big(\ 1+\overline{\varepsilon_{\mathrm{SV}}}\big) \end{eqnarray*}

7 \begin{eqnarray*} {\mathrm{X}}_{\mathrm{ref}}={\mathrm{D}}_{\mathrm{true}}\times \left(\ 1+{\varepsilon}_{\mathrm{WKP}}\right) \end{eqnarray*}

with ${\varepsilon}_{\mathrm{SV}}$ ~ N(0, ${\sigma}_{\mathrm{SV}}$) and ${\varepsilon}_{\mathrm{WKP}}$ ~ N(0, ${\sigma}_{\mathrm{WKP}}$). The noise terms were partially correlated. Substituting into the RD expression:


8 \begin{eqnarray*} \mathrm{RD}\ \left(\%\right)=\left(\frac{{\mathrm{X}}_{\mathrm{SV}}-{\mathrm{X}}_{\mathrm{ref}}}{{\mathrm{X}}_{\mathrm{ref}}}\right)\times 100 \end{eqnarray*}

9 \begin{eqnarray*} =\left(\frac{\ \overline{\varepsilon_{\mathrm{SV}}}-{\varepsilon}_{\mathrm{WKP}}}{1+{\varepsilon}_{\mathrm{WKP}}}\right)\times 100 \end{eqnarray*}

This demonstrates that RD depends only on measurement noise, justifying the use of the standard deviation of RD to assess precision.

### Motivation for using a power model

To describe the relationship between ${\mathrm{SV}}_{\mathrm{mean}}(k)$ and WKP, we define:


10 \begin{eqnarray*} \mathrm{RD}(k)=\left(\frac{{\mathrm{SV}}_{\mathrm{mean}}(k)-\mathrm{WKP}}{\mathrm{WKP}}\right)\times 100 \end{eqnarray*}

We then compute the standard deviation (SD) of RD(*k*) across kidneys:


11 \begin{eqnarray*} \mathrm{sd}\left(\mathrm{RD}(k)\right)=\mathrm{sd}\left(\frac{{\mathrm{SV}}_{\mathrm{mean}}(k)-\mathrm{WKP}}{\mathrm{WKP}}\right) \end{eqnarray*}

The standard deviation was used as a measure of precision. We model this using the power law:


12 \begin{eqnarray*} U(k)=\alpha \times{k}^{-\beta }+\gamma \end{eqnarray*}

This model offers an interpretable structure, where the term $\alpha \times{k}^{-\beta }$ describes the expected improvement in precision with increasing *k;* parameter *β* allows for deviation from ideal $1/\surd k$ scaling, and *γ* reflects the residual imprecision from the WKP estimate. As the number of SVs increases, the mean absorbed dose estimate (${\mathrm{SV}}_{\mathrm{mean}})$ becomes more precise, and the variability in RD between the SV-derived- and WKP-derived doses converges towards a constant value *γ*.

This constant *γ* represents the relative imprecision inherent in the WKP. It includes sources, such as segmentation, PVC, and sampling limitations. That is,


13 \begin{eqnarray*} \gamma \approx \frac{\mathrm{sd}\left({\varepsilon}_{\mathrm{WKP}}\right)}{{\mathrm{D}}_{\mathrm{true}}} \end{eqnarray*}

In practical terms, *γ* is the best achievable relative precision, bounded by WKP noise. This interpretation is valid even without full knowledge of the noise correlation structure, thereby making the model widely applicable.

## Results

**Figure 1:**
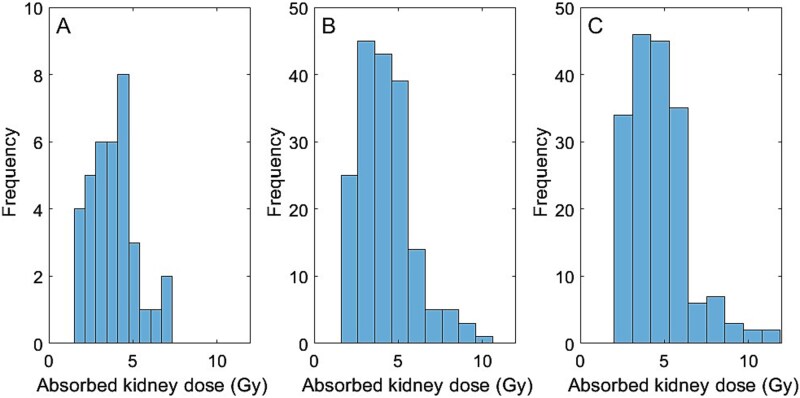
Histograms depicting the distribution of kidney absorbed doses (Gy) estimated across measurements using three methods: A) whole kidney parenchymal (WKP), B) small 2 mL volume (SV2), and C) small 0.6 mL volume (SV0.6), highlighting differences in estimation precision among the methods.

The median and range of absorbed dose estimates in unfiltered images of the 36 kidneys were 3.76 Gy (1.97–7.33) for D_WKP_, 4.03 Gy (2.02–9.83) for D_SV2_, and 4.36 Gy (2.25–11.80) for D_SV0.6_ (Fig. 1). The normalization factor (NF_SV_) for SV_2_ and SV_0.6_ were 1.12 and 1.23, with associated uncertainties (SEMs) of 1.0% and 1.2%, respectively. For filtered images with GFs of 3, 4, 5, and 6 mm, the NF_SV_ for SV_2_ were 1.05, 1.01, 0.96, and 0.91, respectively. The corresponding NF_SV_ for SV_0.6_ were 1.14, 1.09, 1.02, and 0.96, respectively. All NF_SV_ uncertainties were ≤1.2%.

**Figure 2:**
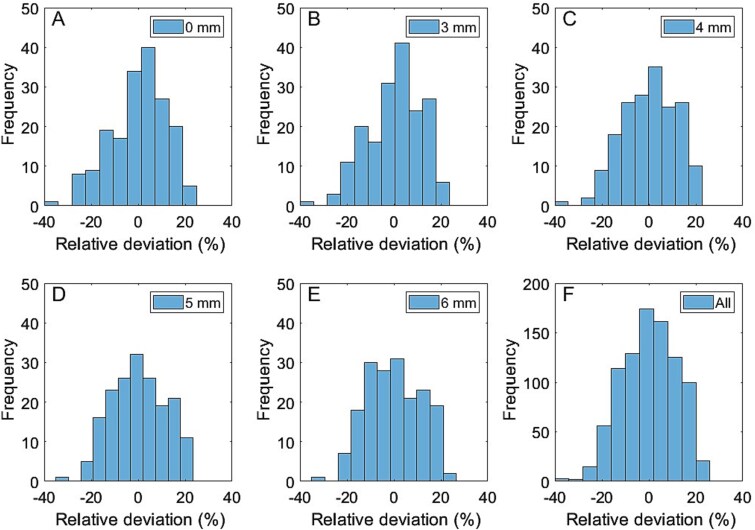
Histograms showing the distribution of relative differences in kidney absorbed dose estimated from single 2 mL small volume (SV2) measurements, illustrating the variability of dose estimates with different Gaussian filter sizes: A) 0 mm, B) 3 mm, C) 4 mm, D) 5 mm, E) 6 mm, and F) combined data for all measurements.

The relative precision for single SV_2_ measurements was 11.82%, 11.41%, 11.38%, 11.29%, and 11.40% for GFs of 0, 3, 4, 5, and 6 mm, and for all measurements combined, respectively (Fig. 2). The corresponding values for SV_0.6_ were 13.13%, 12.30%, 12.11%, 12.07%, and 12.24%. The bias across all measurements was negligible, with values of ≤0.003%.

**Figure 3:**
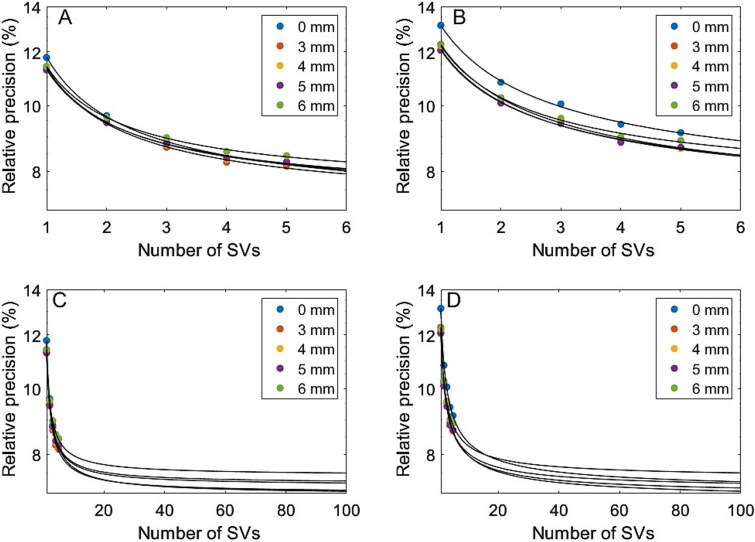
Relative precision in estimated kidney absorbed dose as a function of the number of small volume (SV) measurements, shown for different Gaussian filter sizes: A) and C) correspond to 2mL SV (SV2) measurements, and B) and D) correspond to 0.6 mL SV (SV0.6) measurements, illustrating how precision varies with the number of SVs and applied Gaussian filter, with the x-axis extended to 100 SVs to show extrapolated precision beyond the measured 5 SVs.

Increasing the number of SVs per kidney improved relative precision, as shown in Fig. 3. The power-law model fits the data well, with *R*^2^ values exceeding 0.97. The median relative precision across different GFs was 7.27% (range: 7.05%–7.51%) for SV_2_ and 6.92% (range: 6.81%–7.35%) for SV_0.6_. Statistical testing using the Wilcoxon rank-sum test showed no significant difference in relative precision between the SV_2_ and SV_0.6_ methods (*P* = .15).

## Discussion

This study introduces an empirical power-law model to characterize the relationship between absorbed dose estimates obtained from small VOIs (SVs) and the WKP. By modelling the relative precision of these methods, we aimed to establish a practical framework for assessing the measurement precision of SPECT-based kidney dosimetry.

The accuracy of absorbed dose estimation in molecular radiotherapy (MRT) is influenced by several factors, such as the activity measurement (Bq) using a dose calibrator for image-based absorbed dose estimation, as discussed elsewhere [12]. In individualized patient-specific dosimetry, precise data acquisition, image quantification, organ volume measurements, and optimized curve-fitting for time activity curve estimation have the potential to significantly reduce model-based uncertainties to a value of perhaps ±10%–20% [21].

Manual segmentation of organs on SPECT/CT is time-consuming and subject to interobserver variability, which can also affect AI-based segmentation, as it primarily depends on manual segmentation and the amount of data used to train the network [22]. These volume segmentation errors propagate to other parameters required for estimating the absorbed dose, such as the RC, counts, activity fitting, time-integrated activity, and absorbed dose. Finocchiaro *et al.* [23], analysed the uncertainties in absorbed dose estimation for tumours and demonstrated that volumes are a major source of variability in dosimetry calculations. In cases where the volume of interest was small, the uncertainty in the absorbed dose increased significantly but decreased as the delineated volume increased. The average absorbed dose rate relative uncertainty was reported to be 58%, with a median value of 69%, and a range of 11% to 87% [23].

Our results demonstrate that increasing the number of SVs improves the relative precision of the absorbed dose estimates, which is in line with the theoretical expectation of variance reduction proportional to 1/√k. However, the observed behaviour was more accurately described by a flexible power-law model, in which the fitted exponent *β* deviated slightly from the theoretical ideal. This deviation likely reflects additional sources of variability, such as spatial heterogeneity in the tracer distribution and PVEs, which affect the precision beyond that captured by averaging alone.

The PVE remains a significant challenge in quantitative SPECT imaging because of the limited spatial resolution of SPECT/CT systems, which is influenced by collimator design, detector resolution, and organ size, particularly in radionuclide therapy dosimetry [10]. In structures smaller than three times the full width at half-maximum, PVE leads to activity underestimation or overestimation due to spill-in and spill-out effects [24]. Despite efforts to correct PVE, no standardized method exists, and experimentally derived RCs are often recommended [25]. In [^177^Lu]Lu-DOTATATE therapy, accurate kidney dosimetry is crucial, as the kidneys are considered dose-limiting organs [26]. Studies using 3D-printed patient-specific phantoms have highlighted the impact of PVE on quantitative SPECT/CT imaging and the necessity for correction [27–30]. However, existing phantom studies have limitations in replicating complex intra-renal structures, emphasizing the need for patient-specific PVE assessments to improve the dosimetry accuracy. In this study, we applied patient-specific kidney RCs for absorbed dose estimation to reduce uncertainties in the absorbed dose estimate using the WKP method. Previous studies have shown that using the EANM-recommended RC value of 0.85 instead of patient-specific values, can introduce a 4% uncertainty in absorbed dose estimation [13].

Our findings further emphasize the impact of volume size on percentage variation in absorbed dose calculations using the SV method, specifically for 2 ml, and 0.6 ml volumes. Particular attention was paid to the asymptotic floor parameter *γ*, which represents the residual variability intrinsic to the WKP estimate. Our analysis shows that *γ* remains relatively stable across different Gaussian smoothing levels and SV sizes, reinforcing its interpretation as a surrogate for the minimal achievable excellent fit to the data (*R*^2^ > 0.97) and supporting its suitability for precision modelling in clinical imaging workflows.

Although the SV_2_ and SV_0.6_ methods showed minor differences in normalization factors and precision matrices, no statistically significant difference in relative precision was observed. This finding suggests that smaller SVs, when appropriately normalized, can be used as reliable substitutes for larger ones. This flexibility may be beneficial in clinical settings, where the placement of larger VOIs might be hindered by anatomical constraints or artefacts (e.g. near the kidney edge or in areas of heterogeneous uptake).

This modelling approach provides a quantitative rationale for using multiple SVs to estimate the precision of the WKP-based kidney dosimetry. The power-law model offers a theoretical basis for interpreting the observed variability by capturing how measurement noise scales with the number of SVs. This supports the use of SV sampling not only as a practical alternative to whole-kidney segmentation, but also as a surrogate for estimating the precision of WKP-based absorbed dose calculations in clinical imaging workflows.

The proposed model allows for the estimation of WKP precision from a limited number of SVs, which can aid in standardizing and reporting precision metrics for internal dosimetry. However, this study has several limitations that must be acknowledged. First, the power-law model assumes a smooth, monotonic decrease in variability with increasing *k*, which may not hold in the presence of localized noise or segmentation errors. Second, although *γ* is conceptually linked to WKP-related uncertainty, it may also reflect residual modelling limitations, such as spatial correlations or systematic SV placement bias. Third, the assumption that SV_mean_ approximates a true WKP estimate becomes less valid at a high *k* if the WKP is subject to its own uncertainties, including those introduced by the PVC. Finally, as a data-driven approach, the model is sensitive to outliers and assumptions regarding the independence of the SV and WKP estimates.

Future work should aim to validate this framework in other organs and imaging modalities and in more heterogeneous patient populations. Integrating precision modelling with bias estimation techniques can facilitate a more complete characterization of uncertainty in personalized dosimetry for radionuclide therapy. With the expanding role of MRT and the increasing number of centres performing dosimetry, it is crucial to systematically evaluate and minimize uncertainties in absorbed dose estimations. Accurate activity quantification is essential for individualized treatment planning and optimization. Addressing the uncertainties associated with absorbed dose calculations will improve the precision of dose–response relationships, ultimately leading to more effective and personalized treatment strategies for NET patients receiving [^177^Lu]Lu-DOTATATE therapy.

## Conclusions

This study presents a novel method to quantify the relative precision of SV- and WKP- based dose estimates using an empirical power-law model. The asymptotic floor *γ* serves as a meaningful proxy for the residual WKP imprecision, whereas the *α* and *β* parameters describe the evolution of precision with the number of SVs. Grounded in statistical theory and validated in a clinically realistic setting, this approach offers a flexible and interpretable tool for assessing the SPECT-derived kidney dosimetry precision. However, given the complexity of the noise correlations between the SV and WKP estimates, further validation is warranted before broadly applying this framework to internal dosimetry precision reporting.

## Data Availability

The data supporting the findings of this study are available from the corresponding author upon reasonable request.

## References

[1] Lee A, Chan DL, Wong MH. et al. Systematic review of the role of targeted therapy in metastatic neuroendocrine tumors. Neuroendocrinology 2017;104:209–22. 10.1159/000446115 27082107 PMC5457290

[2] Bodei L, Cremonesi M, Grana CM. et al. Peptide receptor radionuclide therapy with 177Lu-DOTATATE: the IEO phase I-II study. Eur J Nucl Med Mol Imaging 2011;38:2125–35. 10.1007/s00259-011-1902-1 21892623

[3] Paganelli G, Sansovini M, Ambrosetti A. et al. 177 Lu-Dota-octreotate radionuclide therapy of advanced gastrointestinal neuroendocrine tumors: results from a phase II study. Eur J Nucl Med Mol Imaging 2014;41:1845–51. 10.1007/s00259-014-2735-5 24615468

[4] Strosberg J, El-Haddad G, Wolin E. et al. Phase 3 trial of 177Lu-dotatate for midgut neuroendocrine tumors. N Engl J Med 2017;376:125–35. 10.1056/NEJMoa1607427 28076709 PMC5895095

[5] Barone R, Borson-Chazot F, Valkema R. et al. Patient-specific dosimetry in predicting renal toxicity with (90)Y-DOTATOC: relevance of kidney volume and dose rate in finding a dose-effect relationship. J Nucl Med 2005;46:99S–106S 15653658

[6] Sundlöv A, Sjögreen-Gleisner K, Svensson J. et al. Individualised 177Lu-DOTATATE treatment of neuroendocrine tumours based on kidney dosimetry. Eur J Nucl Med Mol Imaging 2017;44:1480–9. 10.1007/s00259-017-3678-4 28331954 PMC5506097

[7] Eberlein U, Cremonesi M, Lassmann M. Individualized dosimetry for theranostics: necessary, nice to have, or counterproductive? J Nucl Med 2017;58:97S–103S. 10.2967/jnumed.116.186841 28864620

[8] Marin G, Vanderlinden B, Karfis I. et al. A dosimetry procedure for organs-at-risk in 177Lu peptide receptor radionuclide therapy of patients with neuroendocrine tumours. Phys Med 2018;56:41–9. 10.1016/j.ejmp.2018.11.001 30527088

[9] Flux GD, Sjogreen Gleisner K, Chiesa C. et al. From fixed activities to personalized treatments in radionuclide therapy: lost in translation? Eur J Nucl Med Mol Imaging 2018;45:152–4. 10.1007/s00259-017-3859-1 29080096 PMC5700228

[10] Ritt P, Vija H, Hornegger J. et al. Absolute quantification in SPECT. Eur J Nucl Med Mol Imaging 2011;38:69–77. 10.1007/s00259-011-1770-8 21484383

[11] Sandström M, Ilan E, Karlberg A. et al. Method dependence, observer variability and kidney volumes in radiation dosimetry of (177)Lu-DOTATATE therapy in patients with neuroendocrine tumours. EJNMMI Phys 2015;2:24. 10.1186/s40658-015-0127-y 26501825 PMC4883125

[12] Gear JI, Cox MG, Gustafsson J. et al. EANM practical guidance on uncertainty analysis for molecular radiotherapy absorbed dose calculations. Eur J Nucl Med Mol Imaging 2018;45:2456–74. 10.1007/s00259-018-4136-7 30218316 PMC6208822

[13] Khan J, Rydèn T, Van Essen M. et al. Dosimetric implications of kidney anatomical volume changes in ^177^Lu-DOTATATE therapy. EJNMMI Phys. 2024;11:71. 10.1186/s40658-024-00672-w 39090481 PMC11294297

[14] Gustafsson J, Brolin G, Cox M. et al. Uncertainty propagation for SPECT/CT-based renal dosimetry in (177)Lu peptide receptor radionuclide therapy. Phys Med Biol 2015;60:8329–46. 10.1088/0031-9155/60/21/8329 26458139

[15] Drake J, Finke A, Ferguson R. Modelling human endurance: power laws vs critical power. bioRxiv . 10.1101/2022.08.31.506028 31 August 2022 PMC10858092 37563307

[16] Clauset A, Shalizi CR, MEJ N. Power-law distributions in empirical data. arXiv preprint arXiv:0706.1062. 2007, https://arxiv.org/abs/0706.1062

[17] Tobias R . Development of methods for analysis and reconstruction of nuclear medicine imaging. Doctoral dissertation. Gothenbrug, Sweden: University of Gothenburg, 2016.

[18] Rydén T, Heydorn Lagerlöf J, Hemmingsson J. et al. Fast GPU-based Monte Carlo code for SPECT/CT reconstructions generates improved 177Lu images. EJNMMI Phys 2018;5:1 10.1186/s40658-017-0201-8 PMC5754277 29302810

[19] Khan J, Rydèn T, Van Essen M. et al. Evaluation of using small volume of interest regions for clinical kidney dosimetry in 177Lu-DOTATATE treatments. Res Sq 2023;12:66. 10.21203/rs.3.rs-2942516/v1 PMC12234931 40624411

[20] Khan J, Rydèn T, van Essen M. et al. Activity concentration estimation in automated kidney segmentation based on convolution neural network method for 177LU–SPECT/CT kidney dosimetry. Radiat Prot Dosim 2021;195:164–71. 10.1093/rpd/ncab079 34080002

[21] Siegel JA, Thomas SR, Stubbs JB. et al. MIRD pamphlet no. 16: techniques for quantitative radiopharmaceutical biodistribution data acquisition and analysis for use in human radiation dose estimates. J Nucl Med 1999;40:37S–61S 10025848

[22] Renard F, Guedria S, De PN. et al. Variability and reproducibility in deep learning for medical image segmentation. Sci Rep 2020;10:1–16 32792540 10.1038/s41598-020-69920-0 PMC7426407

[23] Finocchiaro D, Gear JI, Fioroni F. et al. Uncertainty analysis of tumour absorbed dose calculations in molecular radiotherapy. EJNMMI Phys 2020;7:63. 10.1186/s40658-020-00328-5 33044651 PMC7550507

[24] Dewaraja YK, Frey EC, Sgouros G. et al. 23: quantitative SPECT for patient-specific 3-dimensional dosimetry in internal radionuclide therapy. J Nucl Med 2012;53:1310–25. 10.2967/jnumed.111.100123 22743252 PMC3465844

[25] Ljungberg M, Celler A, Konijnenberg MW. et al. MIRD pamphlet no. 26: joint EANM/MIRD guidelines for quantitative 177Lu SPECT applied for dosimetry of radiopharmaceutical therapy. J Nucl Med 2016;57:151–62. 10.2967/jnumed.115.159012 26471692

[26] Beauregard JM, Hofman MS, Pereira JM. et al. Quantitative (177)Lu SPECT (QSPECT) imaging using a commercially available SPECT/CT system. Cancer Imaging 2011;11:56–66. 10.1102/1470-7330.2011.0012 21684829 PMC3205754

[27] Filippou V, Tsoumpas C. Recent advances on the development of phantoms using 3D printing for imaging with CT, MRI, PET, SPECT, and ultrasound. Med Phys 2018;45:e740–60. 10.1002/mp.13058 29933508 PMC6849595

[28] Woliner-van der Weg W, Deden LN, Meeuwis APW. et al. A 3D-printed anatomical pancreas and kidney phantom for optimizing SPECT/CT reconstruction settings in beta cell imaging using 111In-exendin. EJNMMI Phys 2016;3:29. 10.1186/s40658-016-0165-0 27928774 PMC5143330

[29] Kühnel C, Seifert P, Mulik C. et al. 3D printing of fillable individual thyroid replicas based on nuclear medicine DICOM data used as phantoms for gamma probe calibration. Nuklearmedizin. 2020;59:12–9 31856284 10.1055/a-1070-9874

[30] Tran-Gia J, Salas-Ramirez M, Lassmann M. What you see is not what you get: on the accuracy of voxel-based dosimetry in molecular radiotherapy. J Nucl Med 2020;61:1178–86. 10.2967/jnumed.119.231480 31862802 PMC7413234

